# Dentistry Is to the Eye That Sees It as to That Eye It Seems to Be

**Published:** 1900-04

**Authors:** A. J. Flanagan

**Affiliations:** Springfield, Mass.


### DENTISTRY IS TO THE EYE THAT SEES IT AS TO THAT EYE IT SEEMS TO BE.[1]

BY DR. A. J. FLANAGAN, SPRINGFIELD, MASS.

WHEN the printed notice of this meeting was received by the members of the Institute, and they had read as far as the title of this paper, peculiar thoughts may have emanated from their minds. It is possible that some of those thoughts would not be pleasant, if made audible, and probable that they would not care to repeat them anyway—even to me. My mind travels back a little more than a decade to the city of Philadelphia, and methinks I hear the pleasant voice of good Dr. Garretson saying, "A thing is to the eye that sees it as to that eye it seems to be." Some of my listeners may have had the good fortune to have passed days of studentship under the guidance of the professor; to those this saying will recall the fact that he seldom lectured on any subject without incorporating into it a certain amount of philosophy. The writer has taken the liberty of changing this quotation to suit his fancy. When one cogitates on this, he has come to the conclusion that it is a most apropos title, giving him many strong forts to retire behind in case of controversy. You know there are many abnormal conditions of the eye, and if a person has a myopic, amblyopic, or astigmatic eye his view of dentistry may still be an honest one, even if judged erroneous by others. By the time the last word of this paper is voiced you may have decided that my view must have been through green "goggles."

In summing up the true estimate of any profession or calling, there is one question that should always be asked: What has it done for philanthropy? Philanthropy can justly be said to be love of mankind, and love of mankind is charity. You may ask, What is *true* charity? True charity is that priceless gem by God affixed in the human soul, to measure man's allegiance to the highest and holiest laws of heaven,—that by which the world is made akin. Go where we may in this broad land, we find evidence of charity freely given by medicine. You of the metropolis hear and see its good work almost daily. You see men and women of the highest attainments in medicine and surgery giving freely of their time and pro-

---

[1] Read before The New York Institute of Stomatology, January 2, 1900.

ficiency without pecuniary reward. Perchance you glance your eye down the list of hospital and dispensary staffs: you will find the names of the foremost men in their calling serving there. Now what of our beloved dentistry? We all must admit that dentistry in certain forms is a necessity, and not a luxury; the poor as well as the rich should have its best efforts. After a thorough investigation of this subject, I have yet to find one dental institution or association dispensing true charity. True it is that we have many so-called "college clinics," but it is fair to presume that these cannot be called charitable dental institutions. If we did admit that these were such, how about the staff of operators? Would a medical student be allowed to represent the good charity of medicine? At these dental clinics we see hundreds of dental students operating, but none of the foremost men in that vocation. True it is that the conditions governing medical work are far different from what they would be in dentistry, yet it behooves us to solve this problem. "To whom much is given much is expected." When the epitaph of dentistry is written, may we see emblazoned in lasting device that which is emblematic of charity.

At the present time there is just criticism of our vocation in this one respect. Where there is plenty, charity is a duty, not a courtesy; it is a tribute imposed upon us by heaven, and he is not a good subject who refuses to pay it.

A well-equipped hospital, well managed, is a blessing in any community; you will agree with me when I state that their value to your city has never been justly estimated. What is true of New York City is equally true in a degree of smaller places of residence.

On the staffs of these various institutions are the names of many specialties, save one,—that of stomatology. If its members accomplish good results in every-day practice, why not also in hospitals? Fractures of the maxillæ are considered by many surgeons as difficult to treat. Surgeons of repute are still using the peculiar method, and in extreme cases wire the broken parts. It might be well to state that wiring usually causes a disfigurement. It is my frank opinion that all such cases demand the services of an ingenious dentist. The various forms of interdental splints, correctly applied, always accomplish positive results. Are there not cases where an obturator does greater benefit than an operation for cleft palate? Much is sometimes promised by a surgeon, after an operation of this character, and yet the years only prove it a failure. It is not uncommon to see the use of the hand-drill, chisel, and mallet in cases of oral surgery. It requires a trained hand to rightly use the surgical engine. Such being admitted a fact, and other requirements equal, the hand trained daily in the use of the dental engine should guide the surgical one to success.

In operations of diseased bony structure, how necessary it is to have a fine sense of touch, which can discriminate between normal and abnormal structure. Could certain forms of dyspepsia have their origin in imperfect mastication of food? If this be a fact, what of the people who cannot masticate at all? What more common trouble than that which is ordinarily called facial neuralgia? The fifth pair of nerves has many ramifications, and frequently an obscure source can be traced to a tooth. In a similar case, would it be honest treatment to administer drugs for alleviation?

In lesions of a dental nature why not the services of the specialist? To my mind there is no greater and surer road to a higher appreciation of our calling than by the *entrée* to the hospitals. "By their fruits shall ye know them," is a good biblical saying, and fully as true to-day as in the past.

Apropos to these thoughts is the good we may do for the army and navy. The powers that be firmly maintain that the oral organs of the candidate soldier or sailor shall be in a certain acceptable condition. Such being the case, is it not rather strange that Uncle Sam does not keep them in that condition? It has been my pleasure to meet many men who have been in our government's service, and invariably the men in the lower ranks have had little dental service, other than extraction. The officers have opportunities and privileges in securing dental service. It is not uncommon for them to absent themselves for days to secure the desired service. We have sadly neglected our museum at Washington, and we have thus missed opportunities to present in a tangible form what dentistry has done and is doing for humanity.

In a conversation had with Dr. Donnally, of Washington, he voiced the thought that this neglect might be one of the reasons why we did not secure the desired recognition at the capital. Up to last August there was not even an interdental splint there. Let us demonstrate our ability to do something besides extracting, filling, and inserting teeth; then we may have the desired assistance from the medical fraternity. As our good work goes on, may we bring to use all science and knowledge that will help mankind. Education is but a training of our faculties for a higher and nobler use. Remember that that advancement which ripens but the fruit of individual and class attainment will surely decay and pass back to its elements, selfishness and narrowness.

The *esprit de corps* of any profession has one common source: like unto the ray of light, it starts from a common point, but so diverges that it passes into space, refining and gilding all in an ever-increasing area. Hidden from the eye, within each and every one, is a source of truth for all true advancement; its rays equally brighten and purify all on whom they fall. Can we not call that source ethics?

